# Serological responses to vaccination in children exposed in utero to ustekinumab or vedolizumab: cross-sectional analysis of a prospective multicentre cohort

**DOI:** 10.1007/s00431-024-05683-4

**Published:** 2024-07-18

**Authors:** Katarina Mitrova, Karin Cerna, Kristyna Zdychyncova, Barbora Pipek, Jana Svikova, Petra Minarikova, Miroslava Adamcova, Jan David, Milan Lukas, Dana Duricova

**Affiliations:** 1 IBD Clinical and Research Centre, ISCARE a.s., Prague, Czech Republic; 2 Department of Pediatrics, 2nd Faculty of Medicine, Charles University in Prague and Motol University Hospital, V Uvalu 84, 150 06 Prague, Czech Republic; 3 GENNET s.r.o., Prague, Czech Republic; 4 Digestive Diseases Centre, Hospital AGEL Vitkovice, Ostrava, Czech Republic; 5 2nd Department of Internal Medicine Gastroenterology and Geriatrics, Faculty of Medicine and Dentistry, Palacky University and University Hospital, Olomouc, Czech Republic; 6 Department of Internal Medicine, Jihlava Hospital, Jihlava, Czech Republic; 7 Department of Medicine 1st Faculty of Medicine Charles University and Military Hospital, Military University Hospital Prague, Prague, Czech Republic; 8 Private practice of General Pediatrician, Prague, Czech Republic; 9 Department of Children and Adolescents, Third Faculty of Medicine, Charles University and University Hospital Kralovske Vinohrady, Prague, Czech Republic; 10 Department of Pharmacology, 1st Faculty of Medicine, Charles University, Prague, Czech Republic

**Keywords:** Biologic treatment, Prenatal exposure, Vaccination, Pregnancy, Inflammatory bowel disease

## Abstract

Evidence on serological responses to vaccination in children exposed to ustekinumab (UST) or vedolizumab (VDZ) in utero is lacking. This multicentre prospective study aimed to assess the impact of prenatal exposure to UST or VDZ due to maternal inflammatory bowel disease (IBD) on serological responses to vaccination and other immunological parameters in exposed children. Children aged ≥ 1 year who were exposed in utero to UST or VDZ and completed at least 1-year of mandatory vaccination were included. We assessed the serological response to vaccination (non-live: tetanus, diphtheria, and *Haemophilus influenzae B*; live: mumps, rubella, and measles), whole blood count, and immunoglobulin levels. The control group comprised unexposed children born to mothers without IBD. A total of 23 children (median age, 25 months) exposed to UST (*n* = 13) or VDZ (*n* = 10) and 10 controls (median age, 37 months) were included. The serological response to vaccination was comparable between the UST and VDZ groups and controls, with an adequate serological response rate of ≥ 80%. Only children exposed to UST showed a slightly reduced serological response to mumps (67% vs. 86% in controls), whereas all children exposed to VDZ showed an adequate response. The majority of the exposed children had normal levels of individual immunoglobulin classes, similar to the controls. No severe pathology was observed in any of the children.

*Conclusion*: Despite the limited sample size, our findings suggest that in utero exposure to VDZ or UST does not significantly impair the vaccine response or broader immunological parameters in exposed children.

**Supplementary information:**

The online version contains supplementary material available at 10.1007/s00431-024-05683-4.

**Table:** 

**What is known:** *• Treatment with anti-TNF inhibitors during pregnancy does not appear to affect serologic response to vaccination in exposed children*. *• Evidence on the efficacy of vaccination in children exposed to ustekinumab or vedolizumab in utero is almost lacking*. **What is new:** *• Our findings suggest that in utero exposure to ustekinumab or vedolizumab does not significantly affect the serological responses to common childhood non-live (tetanus, Haemophilus influenzae B, diphtheria) and live vaccines (measles, mumps, rubella)*. *• No major adverse effects on overall immunological health were observed in children exposed in utero to ustekinumab or vedolizumab*.			

## Introduction

A significant proportion of women with inflammatory bowel disease (IBD) are diagnosed at childbearing age, necessitating consideration of the impact of medication during pregnancy. Proper disease control appears to be the most important factor for favourable pregnancy outcomes [1]. This may require intensive anti-inflammatory therapy, including biologic treatment, which has been a matter of concern regarding its safety on the immune system of prenatally exposed children, including adequacy of response to vaccination.

According to published evidence, treatment with anti-TNF inhibitors during pregnancy does not appear to affect the serological response to vaccination in exposed children [2–6]. However, the evidence on efficacy of vaccination given to children exposed to ustekinumab or vedolizumab in utero is sparse. Although data on the serological response to vaccination (except for the oral vaccine in the case of vedolizumab) in adult patients with immune-mediated diseases on vedolizumab or ustekinumab have been favourable [7–9], the results cannot be automatically extrapolated from the adult population to children exposed to these biologics in utero.

The primary objective of this multicentre prospective study was to evaluate the serological response to vaccination in children exposed to ustekinumab or vedolizumab in utero. The secondary objective was to evaluate other immune system parameters in the exposed children.

## Methods

### Study population

This study is a “sub-study” of prospective multicentre project focused on the safety of biologic therapy during pregnancy in women with IBD, including maternal, pregnancy, and children outcome [10]. Women with IBD and their children treated with ustekinumab or vedolizumab during pregnancy at three IBD centres were offered to participate in this study if they met the following criteria: (i) singleton pregnancy ended in live birth over 1 year ago and (ii) children who had completed at least 1 year of mandatory vaccination.

The control group consisted of children of healthy mothers unexposed to biologic therapy recruited either in collaboration with the general paediatrician or were the offspring of medical staff and their relatives. The children in the control group were selected within the age range of the exposed children.

The mandatory vaccination protocol consisted of a hexavalent non-live vaccine (hepatitis B, *Haemophilus influenzae B*, diphtheria, tetanus, pertussis, and inactivated polio) with a standard 3-dose schedule administered within the first year of age. Mandatory vaccination with live vaccines (mumps, rubella, and measles) begins at 13 months of age, with a second dose administered at 5–6 years of age.

### Data collection

Clinical and demographic data of women with IBD, details of biologic and concomitant therapy during pregnancy, and disease activity were obtained from the patients’ medical files by treating gastroenterologists. Information on the perinatal period (gestational age at birth, mode of delivery, birth weight, perinatal complications, and congenital malformation), breastfeeding, and safety of vaccination of both the exposed and control children was obtained from the mother and/or treating paediatrician. A predefined questionnaire was used to collect all the data.

### Laboratory analysis

A single blood sample was collected from each exposed and control child at the time of recruitment. Blood samples were analysed using standard laboratory methods. Complete blood count and parameters of humoral immunity, including serologic response to vaccination (non-live vaccines: tetanus, *Haemophilus influenzae B*, diphtheria, and live vaccines: measles, mumps, rubella) and measurement of serum levels of immunoglobulins (IgG, IgA, IgM, and IgE) were evaluated. Serologic response to vaccination was assessed if ≥ 3 doses of non-live vaccine and ≥ 1 dose of live vaccine were administered, and were considered adequate or inadequate according to the laboratory range (Supplementary Table 1).

Cord plasma from exposed children was analysed for ustekinumab or vedolizumab concentrations as a standard of care at the included centres, as previously described [10]. Drug levels were measured using the Ustekinumab ELISA mAb-based assay [IG-AB121] or Vedolizumab ELISA mAb-based assay [IG-AB116], both manufactured by ImmunoGuide, AybayTech Biotechnology.

### Statistical analysis

Standard descriptive statistics were used, such as absolute numbers and percentages for categorical data, and medians (ranges) for continuous data. Comparisons between the groups were performed using Fisher’s exact test or the Mann-Whitney test. A general linear regression model was used to adjust for age at the time of blood sampling. *p*-value < 0.05 was considered statistically significant. Statistical analysis was performed using the SPSS software [version 17.0, Chicago, Il., USA].

## Results

Forty-seven female patients with forty-nine pregnancies (28 exposed to ustekinumab and 21 exposed to vedolizumab) and children who fulfilled the inclusion criteria were invited to participate in this study. Of these, 23 (49%) women with a total of 24 children agreed to participate. In one child, the blood sample was unsuccessful due to the child's unwillingness to cooperate with blood collection, leaving 23 children for inclusion (13 exposed to ustekinumab and 10 to vedolizumab) (Supplementary Fig. 1). The remaining women who refused to participate in the study either did not want to stress their children with vein punctures or were not interested in the project because of time constraints. The control group consisted of ten unexposed children born to healthy mothers.

The clinical and demographic characteristics of the mothers with IBD and the control mothers are shown in Table 1. All but one woman treated with ustekinumab had Crohn’s disease, whilst the majority (60%) of females treated with vedolizumab had ulcerative colitis. Most women in both groups continued treatment in the third trimester (85% and 80% in the ustekinumab and vedolizumab groups, respectively). Approximately one-third of the patients in both groups were on concomitant thiopurines, and 40% of the women (*n* = 4) receiving vedolizumab also received systemic corticosteroids. In three women, corticosteroids were administered throughout pregnancy (dose 2, 20 mg daily), whilst the fourth patient received high-dose steroids with subsequent tapering between gestational weeks 16 and 26 because of relapse of her ulcerative colitis. The rate of steroid exposure reflects the fact that half of the women on vedolizumab had active disease at any time during pregnancy.

**Table 1:** Clinical and demographic characteristics of mothers

	UST (*n* = 13)	VDZ (*n* = 10)	Con (*n* = 10)	*p*-value UST vs. Con	*p*-value VDZ vs. Con
Age at delivery (years)*	37.2 (21–39)	32 (26–38)	33 (28–39)	0.61	0.50
Smoking during pregnancy (%)	1 (7.7)	0	0	-	-
Mode of delivery (%)				0.67	0.65
Vaginal	7 (54)	5 (50)	7 (70)		
Caesarean section	6 (46)	5 (50)	3 (30)		
Type of IBD (%)			-	-	-
CD	12 (92)	4 (40)			
UC	1 (7.7)	6 (60)			
Disease duration (years)*	11.3 (4.0–22)	12.1 (8.9–20)	-	-	-
CD disease localization# (%)			-	-	-
Ileal	4 (33)	-			
Colonic	3 (25)	-			
Ileocolonic	5 (42)	4 (100)			
Upper	2 (17)	2 (50)			
CD disease behaviour# (%)			-	-	-
Inflammatory	10 (83)	3 (75)			
Structuring	-	1 (25)			
Penetrating	3 (17)	-			
CD—perianal disease# (%)	2 (17)	2 (50)			
UC extension# (%)			-	-	-
Proctitis	-	-			
Left-sided	1 (100)	-			
Extensive	-	6 (100)			
Previous intestinal surgery (%)	3 (23)	1 (10)	-	-	-
Concomitant therapy (%)			-	-	-
None	8 (62)	5 (50)			
Systemic steroids	-	4 (40)			
Thiopurines	5 (39)	3 (30)			
Disease activity			-	-	-
Active**	1 (7.7)	5 (50)			
Remission	12 (92)	5 (50)			

^*^Median (range); **any time during pregnancy; *CD*, Crohn’s disease; *UC*, ulcerative colitis; *UST*, ustekinumab; *VDZ*, vedolizumab; *Con*, controls

^#^Montreal classification

The median ages at blood sampling of the exposed and control children were 25 months (12.8–50.3) and 37.2 months (13.8–57.7), respectively. All but one child in the vedolizumab-treated group were born at term. In this case, the pregnancy was terminated prematurely at 26 weeks of gestation by caesarean section because of severe progressive preeclampsia and pathological placental blood flow. The perinatal data of the exposed and control infants are presented in Table 2. In 12 (92%) children exposed to ustekinumab cord plasma levels of the drug were available with a median level of 11.8 mg/l (0.4–30). The cord plasma levels of vedolizumab were measured in 7 (70%) children with a median level of 5.3 mg/l (1.1–21.3).

**Table 2:** Birth outcomes

	UST (*n* = 13)	VDZ (*n* = 10)	Con (*n* = 10)	*p*-value UST vs. Con	*p*-value VDZ vs. Con
Gestational week of delivery*	39 (37–41)	38.5 (26–41)	39.5 (37–42)	0.61	0.46
Preterm birth (%)	0	1 (10)	0	-	-
Birth weight (grammes)*	3400 (2460–4000)	3075 (650–3755)	3405 (2410–3870)	0.62	0.50
Low birth weight (%)	1 (7.7)	2 (20)	2 (20)	0.56	1.00
Congenital malformation (%)	2 (15) -Hydrocele -Mild hip dysplasia	2 (20) -Cleft lip** -Hypoplasia of kidney	1 (10) -Hypospadias	1.00	1.00
Breastfeeding					
> 2 weeks (%)	11 (85)	9 (90)	10 (100)	1.00	1.00
Length (months) *	12.5 (1–17)	6 (1–15)	7 (2–14)	0.89	0.94

^*^Median (range); **mother had symptomatic SARS-CoV-2 infection during the 1st trimester

*UST*, ustekinumab; *VDZ*, vedolizumab; *Con*, controls

### Serologic response to vaccination

All but one premature child received vaccination according to the national vaccination schedule [11]. In this case, the vaccination schedule was adjusted based on the biological age of the child.

**Fig. 1:**
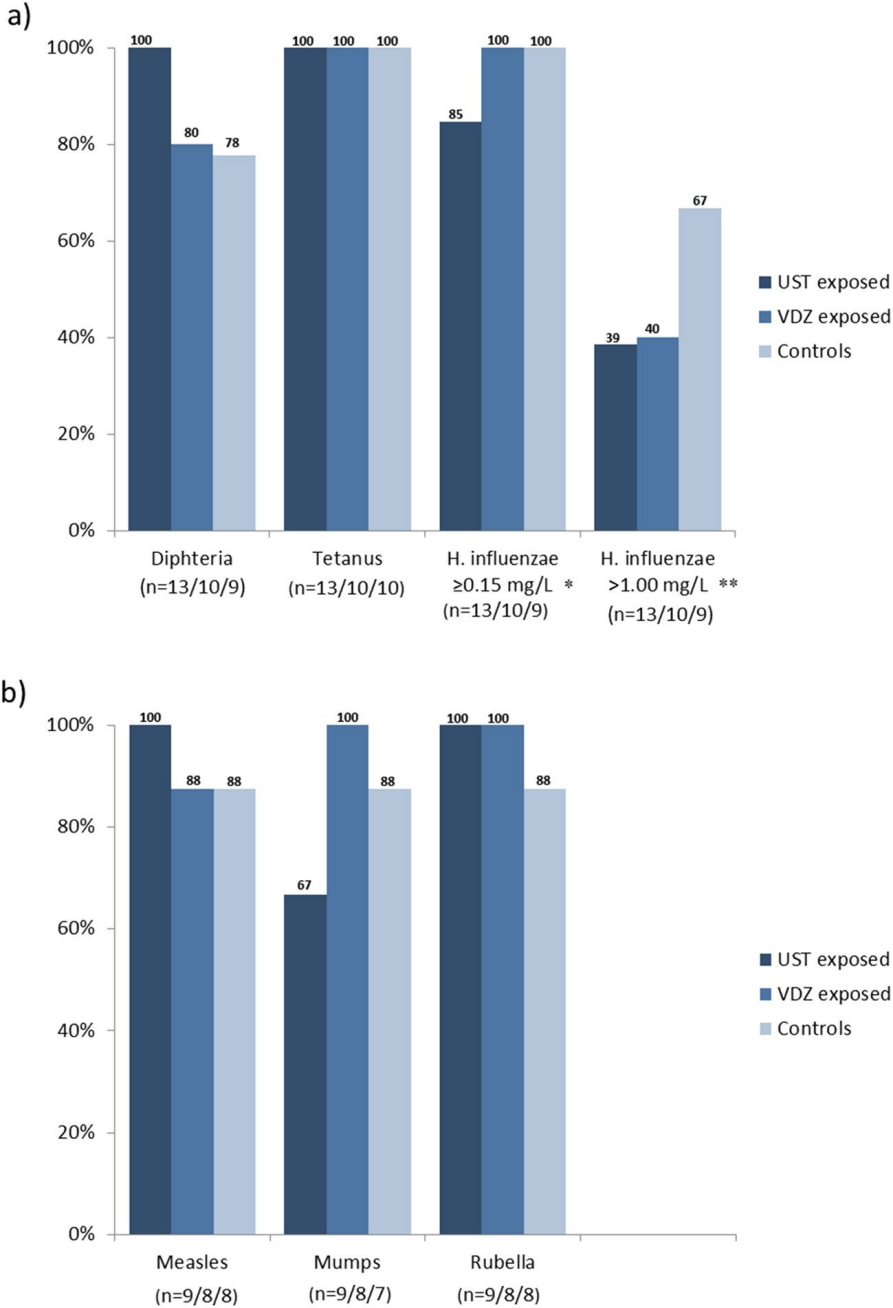
Proportion of children with adequate serologic response to vaccination: **a** non-live vaccines; **b** live vaccines. UST, ustekinumab; VDZ, vedolizumab; * ≥ 0.15 mg/L cut-off for post-vaccination seroprotection; ** > 1 mg/L cut-off for long-term post-vaccination seroprotection

The serological response to non-live vaccination was considered adequate in ≥ 80% of children exposed to ustekinumab or vedolizumab and was comparable to that of controls (Fig. 1).

Two children with ustekinumab exposure showed an inadequate response to *Haemophilus influenzae* vaccination. Both children were born at term, with the last administration of the drug at gestational weeks 27 and 30 and corresponding cord drug levels of 2.8 mg/l and 13 mg/l, respectively. The mother of one child was treated with concomitant thiopurines during pregnancy. However, when assessing long term post-vaccination protection against *Haemophilus influenzae* using a higher cut-off value (> 1.00 mg/L), a numerically lower response rate was observed, with 39% in ustekinumab group and 40% in vedolizumab group versus 67% in healthy controls (compared to controls: *p* = 0.39 for ustekinumab; *p* = 0.37 for vedolizumab).

With vedolizumab, two children (born at term) had insufficient antibody responses to diphtheria. In these children, the last dose of the biologic was administered at weeks 28 and 37 of gestation, resulting in cord blood levels of 1.4 mg/l and 10.4 mg/l, respectively. One child was exposed in utero to concomitant thiopurines and the other was exposed to corticosteroids.

Live vaccines resulted in an adequate serological response in ≥ 80% of the exposed children, which was comparable to that of the control group, except for mumps with numerically lower response rate in children exposed to ustekinumab (67% vs. 88%, *p* = 0.58) (Fig. 1). The mothers of these three children were treated with ustekinumab monotherapy in all three trimesters (the last dose at gestational week 28–35) and delivered at term. In two children, cord blood was available with drug levels of 1.5 mg/l and 10.3 mg/l, respectively.

Adjustment for age at blood sampling did not reveal any significant differences in the serologic response to vaccination between the control and either exposed groups, both for non-live and live vaccines (Supplementary Table 2).

### Safety of vaccination

No clinically relevant adverse reactions to live or non-live vaccinations were observed in either group.

### Other immunological parameters

#### Immunoglobulin levels

Most of the exposed children had normal levels of immunoglobulin classes, and none of the children had severe deficiencies. Three (23%) children exposed to ustekinumab had lower immunoglobulin levels (below the stated laboratory cut-offs for the normal range): three had lower IgA levels (0.20 g/l, 0.31 g/l [cut-off 0.33]; 0.5 g/l [cut-off 0.66]), whilst one had lower IgG (4.69 g/l, cut-off 5.53) and IgM levels (0.36 g/l, cut-off 0.47). Serologic response to vaccination in this child was adequate for diphtheria and tetanus and inadequate for haemophilus. Regarding vedolizumab, one child had lower levels of IgA (< 0.25 g/l, cut-off 0.33), whereas the other child with premature delivery had lower levels of IgA (0.12 g/l, cut-off 0.33), IgG (3.15 g/l, cut-off 5.53), and IgM (0.39 g/l, cut-off 0.47). Despite this, the child developed seroprotective antibodies against all non-live vaccines. In the control group, two children had lower levels of immunoglobulins, one in the IgA and the other in the IgG class. Both children showed an adequate response to the vaccination. Elevated levels of IgE were measured in one and two children exposed to ustekinumab and vedolizumab, respectively, and in one control child. Table 3 displays the serologic response to non-live vaccination with respect to immunoglobulin levels.

**Table 3:** Proportion of children with seroprotective antibody titres to non-live vaccines stratified by immunoglobulin (Ig) levels

Ustekinumab *n* (%)	Low Ig# *n* = 3 (23.1) IgA + IgG + IgM: *n* = 1; IgA: *n* = 2	Normal Ig *n* = 10 (76.9) -
Cord drug levels (mg/l)	*n* = 3: 0.4; 13.0; 13.2	*n* = 9: median 2.8 (range 1.5–30)
Seroprotective titres (%)		
Diphtheria	3/3 (100)	10/10 (100)
Tetanus	3/3 (100)	10/10 (100)
HiB ≥ 0.15	2/3 (66.7)	9/10 (90)
HiB > 1.00	1/3 (33.3)	4/10 (40)
Vedolizumab *n* (%)	Low Ig# *n* = 2 (20) IgA + IgG + IgM: *n* = 1; IgA: *n* = 1	Normal Ig *n* = 8 (80) -
Cord drug levels (mg/l)	*n* = 1: 21.3	*n* = 6: median 3.6 (range 1.1–10.4)
Seroprotective titres		
Diphtheria	2/2 (100)	6/8 (75)
Tetanus	2/2 (100)	8/8 (100)
HiB ≥ 0.15	2/2 (100)	8/8 (100)
HiB > 1.00	0	4/8 (50)
Controls* *n* (%)	Low Ig *n* = 2 (20) IgA: *n* = 1; IgG: *n* = 1	Normal Ig *n* = 7 (70) -
Seroprotective titres		
Diphtheria	2/2 (100)	4/6 (66.7)
Tetanus	2/2 (100)	7/7 (100)
HiB ≥ 0.15**	2/2 (100)	6/6 (100)
HiB > 1.00**	1/2 (50)	5/6 (83.3)

*HiB*, *Haemophilus influenzae B*

^#^Low Ig, below the normal laboratory range

^*^1 child did not have analyses of immunoglobulin levels

^**^ ≥ 0.15 mg/L cut-off for post-vaccination seroprotection; > 1 mg/L cut-off for long-term post-vaccination seroprotection

#### Blood count

One child 26 months old who was exposed to ustekinumab monotherapy had an elevated platelet count (606 × 10^9^/L) with no other pathology in the blood count. No clinically relevant abnormalities were found in either the exposed or control groups.

## Discussion

This multicentre prospective study provides crucial insights into the immunological safety of ustekinumab and vedolizumab during pregnancy. Our data demonstrate that in utero exposure to ustekinumab or vedolizumab does not significantly diminish serological responses to routine childhood vaccinations. The adequate immunological responses observed in our cohort suggests that these biologics, when used during pregnancy, do not adversely affect the developing immune system of the foetus in a clinically significant manner.

Patients with IBD receiving immunosuppressive and/or biologic (anti-TNF) therapy have been reported to have an impaired post-vaccination serologic response to several vaccines [12–16]. Therefore, there have been concerns regarding the adequacy of serologic response to vaccination in children exposed to biologic preparations in utero. Nevertheless, several previous studies have demonstrated adequate serological responses to vaccination in children with prenatal exposure to anti-TNF preparations [3, 6].

However, in the era of newer biologics with different mechanisms of action (e.g., ustekinumab and vedolizumab), new safety concerns have arisen [10, 17–25].

Evidence from studies in adult patients with IBD suggests that vaccination is safe and effective in patients treated with vedolizumab or ustekinumab (except for oral vaccines in vedolizumab) [7–9]. However, these data cannot be automatically extrapolated to children with prenatal exposure to these agents. The main reason for this is the immaturity of their immune system, which is quite different and still develops during the first years of life and could potentially be affected by drug exposure. It is known that ustekinumab can cross the placenta with a transfer pattern similar to that described for anti-TNF agents, but its postnatal clearance seems to be faster (median of 9 weeks, complete by 20 weeks after birth) and even faster for vedolizumab (mean of 3.8 weeks, complete by 6 months) [25, 26]. This may indicate a lower impact of ustekinumab and vedolizumab on serological post-vaccination response. However, it must be kept in mind that the serologic response to vaccination and other immune system functions may be influenced by the drug’s impact on developing the immune system during the antenatal period, and not only by the presence of drug levels after birth. To the best of our knowledge, the only evidence on the immune response to vaccination in children exposed to new biologics comes from a recent study by Beaulieu et al., who reported a normal antibody response to *Haemophilus influenzae B* and tetanus vaccination in two individuals exposed in utero to ustekinumab and one to vedolizumab [4]. Our study confirms this finding in a larger group of children. The majority of our children (≥ 80%) had adequate responses to both non-live (diphtheria, tetanus, and *Haemophilus influenzae*) and live (measles, mumps, and rubella) vaccines, as observed in the control group. This was true for all but two vaccines: *Haemophilus influenzae* and mumps.

For *Haemophilus influenzae*, using a higher cut-off value (> 1.00 mg/L, reflecting long-term protection), an adequate serologic response was observed in only approximately 40% of exposed children and 67% of healthy controls. However, using a lower cut-off value (≥ 0.15 mg/L), which is commonly used in other studies to assess post-vaccination seroprotection against *Haemophilus influenzae*, an adequate response was observed in 85% of children exposed to ustekinumab and 100% of children exposed to vedolizumab or the control group. Currently, it is not clear which cut-offs are appropriate, as different cut-offs have been used in studies assessing serological response to this vaccine [3, 27–30]. Considering the potential risk factors for low serologic response after HiB vaccination (using a higher cut-off) in our cohort, such as maternal disease activity during pregnancy and concomitant corticosteroid or thiopurine use, no such signal was observed (data not shown). Of note, our previous study and the PIANO study of children with anti-TNF exposure in utero also demonstrated a lower serologic response to Haemophilus vaccine (adequate seroprotection below 75%), but this was also the case in the control group of unexposed children [3, 4]. We can only speculate whether the type of vaccine may also play a role, as the stimulation of the immune system may differ between vaccines.

A somewhat lower rate of adequate serology was also observed after mumps vaccination in the ustekinumab group. Most probably, this finding just reflects the limited sample size and further studies are needed to confirm this.

We did not find any pathology in the blood count, except for one child with significant thrombocytosis in the ustekinumab group. When basic immunological parameters, such as total serum immunoglobulin levels, were evaluated, no clinically significant pathology or differences were found between the exposed children and healthy controls. Only three children exposed to ustekinumab and two exposed to vedolizumab had lower total IgA antibody levels, but no severe IgA hypogammaglobulinemia was observed. Similar findings were observed in a recent study from Canada, which primarily evaluated the safety of the rotavirus vaccine in children exposed to biologics in utero. Amongst 191 children exposed to biologic treatment, no clinically significant abnormalities in lymphocyte subsets, quantitative immunoglobulins, or mitogen responses were identified. Similarly, no severe adverse events were reported after rotavirus vaccine administration [17].

Our study has several limitations. The biggest limitation is the small sample size, which precludes us from drawing firm conclusions. Slightly more than half of the women refused to participate mainly because they considered vein puncture too invasive for their children. Nevertheless, to date, it is still the largest documented cohort focused on the efficacy of vaccination in children exposed to ustekinumab or vedolizumab in utero. The small sample size also limited us from conducting further analyses, for example, regarding the impact of cord plasma levels or concomitant maternal immunosuppression. The other limitation is the different ages of the exposed and control groups at blood sampling (median age 25 vs. 37 months, respectively), despite our intention to include a control group of similar age to the exposed group. Thus, the theoretical impact of this difference on the serologic results cannot be excluded.

In conclusion, our research contributes valuable data to the limited body of knowledge regarding the effects of in utero exposure to ustekinumab and vedolizumab on children’s immune responses. Although our study was limited by its sample size, the findings suggest no major adverse effects on vaccine responses or overall immunological health in these children. This information is pivotal for guiding treatment decisions in pregnant women with IBD, and for a broader field of clinical immunology.

## Supplementary information

ESM 1 (PNG 331 kb)Supplementary Figure 1. Flowchart of children recruitment. *two children were born to one woman

High Resolution Image (TIFF 97 kb)

ESM 2 (DOCX 15 kb)

ESM 3 (DOCX 14 kb)

## Data Availability

The data underlying this article will be shared upon reasonable request by the corresponding author.

## Abbreviations

ELISA: Enzyme-linked immunosorbent assay
Hib: *Haemophilus influenzae B*
IBD: Inflammatory bowel disease
Ig: Immunoglobulin
UST: Ustekinumab
TNF: Tumour necrosis factor
VDZ: Vedolizumab
