# Supplementary figures and images for: Serological responses to vaccination in children exposed in utero to ustekinumab or vedolizumab: cross-sectional analysis of a prospective multicentre cohort
Source: Eur J Pediatr. 2024 Jul 18;183(10):4243–51. doi: 10.1007/s00431-024-05683-4 (PMC11413139; doi:10.1007/s00431-024-05683-4)

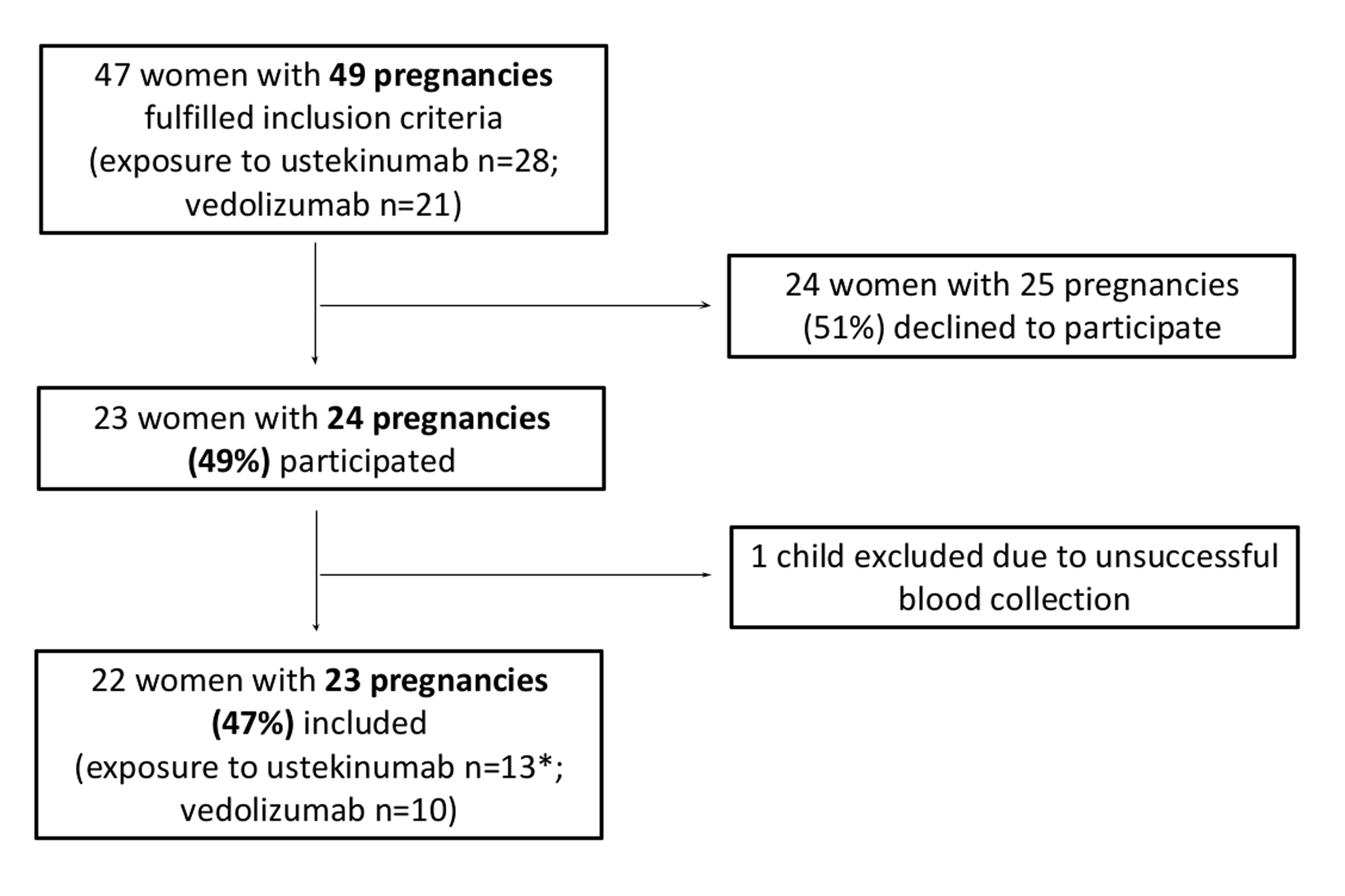

Supplement: Supplementary file 1 — (PNG 331 kb)Supplementary Figure 1. Flowchart of children recruitment. *two children were born to one woman [file 431_2024_5683_Fig2_ESM.png]

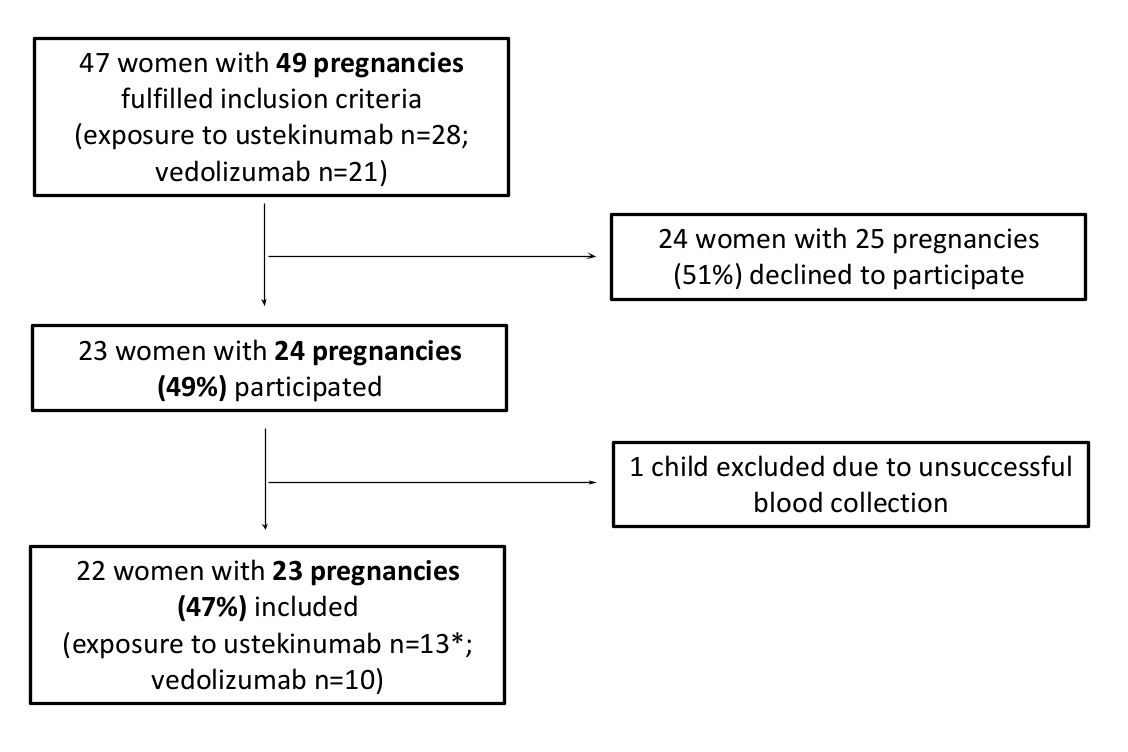

Supplement: Supplementary file 2 — High Resolution Image (TIFF 97 kb) [file 431_2024_5683_MOESM1_ESM.tiff]
