# Supplementary material for: Serological responses to vaccination in children exposed in utero to ustekinumab or vedolizumab: cross-sectional analysis of a prospective multicentre cohort
Source: Eur J Pediatr. 2024 Jul 18;183(10):4243–51. doi: 10.1007/s00431-024-05683-4 (PMC11413139; doi:10.1007/s00431-024-05683-4)
Supplement: Supplementary file 3 — (DOCX 15 kb) [file 431_2024_5683_MOESM2_ESM.docx]

**Supplementary Table 1.** Laboratory values for adequate serologic responses to vaccination

|  | Titre cut-off |
| --- | --- |
| **Non-live vaccines** |  |
| Diphtheria | > 0.1 IU/mL |
| Tetanus | ≥ 0.1 IU/mL |
| *Haemophilus influenzae*   - Serologic response - Long-term serologic response | ≥ 0.15 mg/L  > 1.00 mg/L |
| **Live vaccines** |  |
| Measles | >13.5 AU/ml or >200 mIU/ml |
| Mumps | >9 AU/ml or >22 RU/ml |
| Rubella | >11 IU/ml |
