# Supplementary material for: Serological responses to vaccination in children exposed in utero to ustekinumab or vedolizumab: cross-sectional analysis of a prospective multicentre cohort
Source: Eur J Pediatr. 2024 Jul 18;183(10):4243–51. doi: 10.1007/s00431-024-05683-4 (PMC11413139; doi:10.1007/s00431-024-05683-4)
Supplement: Supplementary file 4 — (DOCX 14 kb) [file 431_2024_5683_MOESM3_ESM.docx]

**Supplementary Table 2.** Adequate serologic response to vaccination – comparison to control group adjusted by age at blood sampling (generalized linear regression model)

|  | aOR (95% CI) |
| --- | --- |
| **Ustekinumab exposure** |  |
| - Tetanus* | - |
| - Diphtheria | Inf. (0-NA) |
| - HiB cut-off ≥15 mg/L | 0 (NA-Inf.) |
| - HiB cut-off >1 mg/L | 0.24 (0.03-1.63) |
| - Mumps | 0.44 (0.01-6.67) |
| - Measles | Inf. (0-NA) |
| - Rubella | Inf. (0-NA) |
| **Vedolizumab exposure** |  |
| - Tetanus* | - |
| - Diphtheria | 0.49 (0.02 - 7.09) |
| - HiB cut-off ≥15 mg/L* | - |
| - HiB cut-off >1 mg/L | 0.35 (0.05 - 2.33) |
| - Mumps | Inf. (0-NA) |
| - Measles | 1.17 (0.03-42.4) |
| - Rubella | Inf. (0-NA) |

aOR – adjusted odds ratio, CI – confidence interval; HiB – *Haemophilus influenza B*

*Analyses not meaningful due to 100% serologic response in both exposed and control group

NA – analysis not valid due to low numbers
